# *Xenopus laevis* RIC-3 enhances the functional expression of the *C. elegans* homomeric nicotinic receptor, ACR-16, in *Xenopus* oocytes

**DOI:** 10.1111/jnc.12013

**Published:** 2012-10-10

**Authors:** Hayley M Bennett, Kristin Lees, Kate M Harper, Andrew K Jones, David B Sattelle, Susan Wonnacott, Adrian J Wolstenholme

**Affiliations:** 1 Department of Infectious Diseases and Center for Tropical & Emerging Global Diseases, University of GeorgiaAthens, GA, USA; 2 Department of Biology & Biochemistry, University of BathBath, UK; 3 Faculty of Life Sciences, University of ManchesterManchester, UK; 4 Department of Biological and Medical Sciences, Oxford Brookes UniversityOxford, UK

**Keywords:** chaperone, nicotinic acetylcholine receptor, whole-cell voltage clamp, *Xenopus* oocyte

## Abstract

RIC-3 enhances the functional expression of certain nicotinic acetylcholine receptors (nAChRs) in vertebrates and invertebrates and increases the availability of functional receptors in cultured cells and *Xenopus laevis* oocytes. Maximal activity of RIC-3 may be cell-type dependent, so neither mammalian nor invertebrate proteins is optimal in amphibian oocytes. We cloned the *X. laevis ric-3* cDNA and tested the frog protein in oocyte expression studies. *X. laevis* RIC-3 shares 52% amino acid identity with human RIC-3 and only 17% with that of *Caenorhabditis elegans*. We used the *C. elegans* nicotinic receptor, ACR-16, to compare the ability of RIC-3 from three species to enhance receptor expression. In the absence of RIC-3, the proportion of oocytes expressing detectable nAChRs was greatly reduced. Varying the ratio of *acr-16* to *X. laevis ric-3* cRNAs injected into oocytes had little impact on the total cell current. When *X. laevis*, human or *C. elegans ric-3* cRNAs were co-injected with *acr-16* cRNA (1 : 1 ratio), 100 μM acetylcholine induced larger currents in oocytes expressing *X. laevis* RIC-3 compared with its orthologues. This provides further evidence for a species-specific component of RIC-3 activity, and suggests that *X. laevis* RIC-3 is useful for enhancing the expression of invertebrate nAChRs in *X. laevis* oocytes.

The *Resistance-to-cholinesterase 3* (*ric-3*) gene was first identified in *Caenorhabditis elegans* (Nguyen *et al*. 1995; Miller *et al*. 1996), and it has subsequently been demonstrated that RIC-3 can influence the maturation of ionotropic receptors, in particular 5-HT_3_ receptors (5-HT_3_Rs) and nicotinic acetylcholine receptors (nAChRs), found in the neurones and musculature of both vertebrate and invertebrate species (Millar 2008; Treinin 2008; Valles and Barrantes 2012). The ability of RIC-3 to increase the availability of functional receptors in expression studies has been reported for mammalian (Castillo *et al*. 2005; Cheng *et al*. 2005; Lansdell *et al*. 2005, 2008; Williams *et al*. 2005; Castelan *et al*. 2008; Roncarati *et al*. 2008; Seredenina *et al*. 2008; Li *et al*. 2009; Valles *et al*. 2009; Wang *et al*. 2009) and insect cell culture (Lansdell *et al*. 2008), and in the *Xenopus laevis* oocyte expression system (Halevi *et al*. 2002, 2003; Cohen Ben-Ami *et al*. 2005, 2009; Williams *et al*. 2005; Castelan *et al*. 2008; Biala *et al*. 2009; Sattelle *et al*. 2009). Some cell lines are non-permissive to nAChR expression in the absence of RIC-3 (Li *et al*. 2009). RIC-3 is also capable of altering the stoichiometry and properties of some nAChRs, as in the case of the *C. elegans* DEG-3/DES-2 receptor (Cohen Ben-Ami *et al*. 2005), and in some cases, it appears to suppress functional expression of nAChRs (Halevi *et al*. 2003; Castelan *et al*. 2008), 5-HT_3_Rs (Halevi *et al*. 2003; Castillo *et al*. 2005; Cheng *et al*. 2007) and α7 nAChR/5-HT_3_R chimaeras (Castillo *et al*. 2005; Gee *et al*. 2007).

RIC-3 is an integral membrane protein with two membrane-spanning domains, TM1 and TM2, the second of which is well conserved between species and has been identified as both necessary and sufficient for receptor modulation (Cohen Ben-Ami *et al*. 2005; Biala *et al*. 2009). Studies in *Xenopus* oocytes with the homomeric ACR-16 receptor from *C. elegans* suggest a conserved TM2 domain is crucial for enhancing expression levels (Cohen Ben-Ami *et al*. 2009). TM1 may be a cleavable signal sequence in human RIC-3 (Cheng *et al*. 2007). On the C-terminal end, the TM domains are followed by one or two coiled-coil domains, which are suggested to be responsible for some of the subunit specificity (Halevi *et al*. 2003; Biala *et al*. 2009). Alternatively spliced transcripts, some of which do not encode the coiled-coil regions, have been suggested as a means by which the protein may exert even more control over subtypes of nAChRs (Halevi *et al*. 2003). A recent model suggests that the coiled-coil domains of mammalian RIC-3 do not bind α7 nAChR directly, but may be part of a homotypic assembly method, bringing multiple subunits, each bound to RIC-3 via the transmembrane region, together (Wang *et al*. 2009).

The idea that the function of RIC-3 may vary in different cellular environments was first raised by Cheng *et al*. (2007) in discussing how the effect of RIC-3 on receptor expression can be opposite in mammalian cells versus amphibian oocytes. Lansdell *et al*. (2008) took this further and demonstrated that *Drosophila* RIC-3 enhanced nAChR expression to a greater extent in a *Drosophila* cell line than in a human one, and that human RIC-3 was more effective at enhancing nAChR expression in human cells compared with *Drosophila* cells. Studies using *Xenopus* oocytes have so far only utilized *C. elegans*, *D. melanogaster* or human RIC-3. Therefore, we have cloned the *Xenopus laevis ric-3* cDNA to investigate whether this could be a useful tool for expression studies and screening of nAChRs in oocytes, particularly in respect of invertebrate nAChR, as *in vitro* expression of these nAChRs is challenging (Millar and Lansdell 2010).

Invertebrate nAChR are of medical and economical importance as targets of many important drugs that act against nematode and insect parasites, vectors and pests (Lees *et al*. 2012). Nematodes express a large number of different nAChR subunits (Mongan *et al*. 1998), including those making up the well-characterized levamisole-sensitive (L-type) and nicotine-sensitive (N-type) receptors (Lees *et al*. 2012) as well as those more recently exploited as novel targets for drugs such as monepantel (Kaminsky *et al*. 2008). Co-expression of chaperones, including RIC-3, along with the receptor subunits is required for efficient channel formation, a prerequisite for functional studies (Halevi *et al*. 2002; Boulin *et al*. 2008). We are using the homomeric nAChR encoded by the *Caenorhabditis elegans acr-16* gene (Raymond *et al*. 2000) to explore the development of improved systems for the expression of nematode nAChR. Co-expression of *C. elegans* ACR-16 with RIC-3 produces robust currents (Biala *et al*. 2009; Cohen Ben-Ami *et al*. 2009; Sattelle *et al*. 2009), and offers a suitable experimental system for comparing RIC-3 from three different species (*X. laevis*, *C. elegans* and human). Evidence that RIC-3 itself can be regulated by other proteins *in vivo* (Shteingauz *et al*. 2009) has raised the question as to whether an excess of RIC-3 may be deleterious to nAChR expression. In this study, we use differing ratios of the *X. laevis ric-3* and *C. elegans* nAChR subunit *acr-16* mRNA to address this issue, and compare the results obtained with the nematode receptor to those from oocytes expressing the human α7 nAChR (Peng *et al*. 1994).

## Materials and methods

### Cloning of *X. laevis ric-3* cDNA

One adult *X. laevis* was terminally anaesthetised using a solution of 0.2% (w/v) benzocaine in tap water and the heart excised. Within 15 min, the brain and spinal cord were removed using an outlined dissection technique (Rowell, 1953), then placed in a 1.5 mL microcentrifuge tube and snap frozen in liquid nitrogen, then stored at −80°C. After thawing, the tissue was placed, along with 2 mL of Trizol® reagent (Invitrogen, Carlsbad, CA, USA), in a 10 mL borosilicate glass homogenizer tube (Jencons, Leighton Buzzard, UK) pre-cleaned with RNase-Away™ (Invitrogen) and the tissue mechanically pulped using the accompanying pestle. A phenol:chloroform (1 : 1) extraction was used to isolate the RNA, which was then reversed transcribed into cDNA and used as the template for PCR amplification. Primer sequences for PCR were based on the *X. tropicalis ric-3* sequence (NCBI accession number BC118843); the forward sequence was ATGGCTCTGTCCGCTGTCCA and the reverse ATGTAGCAATCAGTACACAATGC. The resulting 1117bp product was cloned into the pGEM®–T Easy (Promega, Madison, WI, USA) and the insert sequenced.

### Bioinformatics

Sequences were translated using an online Expasy tool (http://expasy.org/tools/dna.html). Translated sequences were interrogated for transmembrane regions and coiled-coil domains using two servers: TMPred (http://www.ch.embnet.org/software/TMPRED_form.html) and COIL (http://www.ch.embnet.org/software/COILS_form.htm). Signal peptide sequences were predicted using the SignalP 3.0 Server (http://www.cbs.dtu.dk/services/SignalP/). ClustalW2 was used for alignments (http://www.ebi.ac.uk/Tools/msa/clustalw2/).

### Expression in *Xenopus* oocytes

RNA was prepared from plasmid DNA linearized using NcoI (*X. laevis ric-3*), NotI (*C. elegans* and human *ric-3*), ApaI (*acr-16*) or Xba-1 (human α7) and synthesized by the T7 (pCDNA3.1) or SP6 (pGEM®-T Easy) mMessage mMachine kits (Ambion, Austin, TX, USA). The *C. elegans ric-3* cDNA used was that described in Sattelle *et al*. (2009); the human *ric-3* cDNA encoded variant 1, isoform a. All the cDNA clones were sequenced prior to use to confirm that they encoded a functional protein. The human Stage V and stage VI oocytes were selected from *X. laevis* ovaries (NASCO, Fort Atkinson, WI, USA) treated with 2.5 mg/mL collagenase for 30 min, washed, then manually defolliculated using fine forceps. The oocytes were kept in chilled Standard Oocyte Saline (SOS) (pH 7.5, 100 mM NaCl, 1.0 mM MgCl_2_, 5.0 mM HEPES, 2.0 mM KCl, 1.8 mM CaCl_2_) before being injected with a Nanoject 3-00-203-X (Drummond, Broomall, PA, USA). In experiments where the ratio of *ric-3* cRNA:*acr-16* cRNA was varied, oocytes were injected with 50 ng of *acr-16* mRNA with either 200 ng (1 : 4), 50 ng (1 : 1), 12.5 ng (4 : 1) or no (1 : 0) *X. laevis ric-3*cRNA. In experiments comparing RIC-3 from the three different species (human, *C. elegans* and *X. laevis*), 50 nL of 1 μg/μL cRNA was injected, pre-mixed at a 1 : 1 ratio. After injection, oocytes were incubated 48–72 h at 18°C in a modified SOS solution containing 2.5 mM sodium pyruvate, 100 U/mL penicillin and 100 μg/mL streptomycin.

Oocytes were transferred to an oocyte recording chamber and impaled with two mounted glass microelectrodes (3 M KCl and resistance between 0.5 and 5 MΩ in SOS). The membrane potential was clamped with a GeneClamp 500 Amplifier (Molecular Devices, Sunnyvale, CA, USA) at −100 mV and a resting current below 1 μA. SOS solution was applied at a rate of 3 mL/min, a Minipuls (Gilson, Middleton, WI, USA) peristaltic pump was to control chamber volume. Compounds [acetylcholine (ACh) and nicotine] were prepared as fresh solutions daily and bath applied. All recordings were made in the presence of 0.5 μM atropine to prevent activation of endogenous muscarinic AChRs in the oocytes (Davidson *et al*. 1991). A Digidata 1322 (Molecular Devices) was used to digitize the signal and communicate via SCSI to a desktop PC. Recordings were acquired at (1 kHz) and analysed using pClamp9 software (Molecular Devices).

### Data analysis

Individual current inputs were analysed using Clampfit9 (Molecular Devices). EC_50_ values and Hill slopes were determined using non-linear regression on normalized data (100 μM ACh as maximal response) using GraphPad Prism® software (GraphPad Software Inc., San Diego, CA, USA).

## Results

### Molecular cloning of *Xenopus laevis ric-3*

**Fig. 1:**
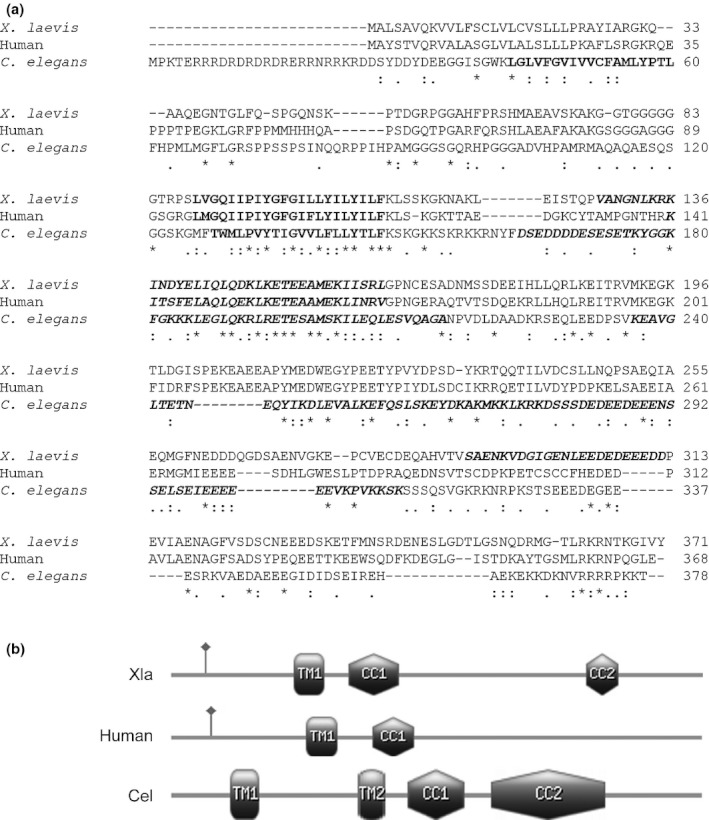
The predicted amino acid sequence and domain structure of *X. laevis* RIC-3. (a) Alignment of translated sequences for *X. laevis*, *C. kelegans* and human RIC-3 showing first and second transmembrane (TM) regions (bold) predicted by TMPred; and the first (bold, highlighted light grey) and second (bold, highlighted dark grey) coiled-coil (CC) domains predicted by COILS. (b) Schematic representation of the domains found in RIC-3 from the three species.

Polymerase chain reaction reactions on *X. laevis* cDNA using primers based on the *ric-3* sequence of *X. tropicalis* resulted in a 1117 bp product that was cloned and sequenced. A BLASTP search using this sequence, translated into protein, showed that it was very similar to RIC-3 from several species and had the highest identity to the full length predicted polypeptide from *X. tropicalis*. Virtual translation of the cDNA sequence and analysis of the resultant polypeptide sequence revealed that two membrane-spanning regions were predicted by TMPred; the first of these is likely to be a signal peptide (Fig. 1). The predicted polypeptide sequence also contained two predicted coiled-coil domains (Fig. 1) and as such, can be considered equivalent to full length *C. elegans* RIC-3, rather than the truncated form reported by Halevi *et al*. (2003). The sequence was deposited in the NCBI sequence database under Accession Number ACF74450.1. Alignment of the translated *Xenopus* RIC-3 sequence with those from mammals and invertebrates showed 52% identity to the human protein, but only 17% with that from *C. elegans* (Table 1), despite human RIC-3 lacking one of the coiled-coil domains. The second membrane-spanning regions are especially well conserved, with the *X. laevis* and human RIC-3 sharing over 90% sequence identity in this region.

**Table 1:** Amino acid identity between the predicted domains (Fig. 1B) of *X. laevis* RIC-3 and the human and *C. elegans* orthologues

Domain	*C. elegans*	Human
TM1	36% (TM2)	91%
CC1	37%	68%
CC2	22%	N/A
Full-length	17%	52%

The TM2 domain of the *C. elegans* RIC-3 is likely to be functionally equivalent to the TM1 domain of the human and *X. laevis* proteins.

### The effect of *X. laevis ric-3* on the expression of ACR-16 in *X. laevis* oocytes

**Fig. 2:**
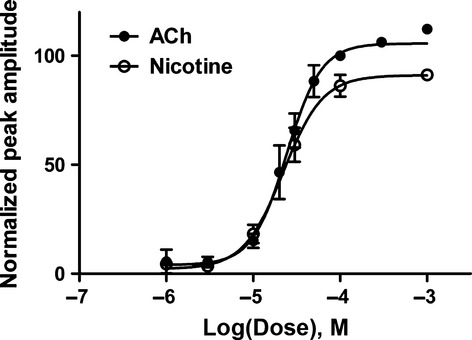
Concentration-response curves for ACR-16 co-expressed with *X. laevis* RIC-3. The mean peak current amplitude [normalized to 100 μM acetylcholine (ACh)] was recorded from voltage clamped oocytes 48 h post-injection with 50 ng *acr-16*/*Xenopus ric-3* cRNA at a 1 : 1 ratio (*n* = 8, *n* = 4). ACh (closed circles) EC_50_ = 24 μM (95% CI = 19–30 μM), Hill Slope = 2.177. Nicotine (open circles) EC_50_ = 22 μM (95% CI = 15–33 μM), Hill Slope = 1.906.

**Fig. 3:**
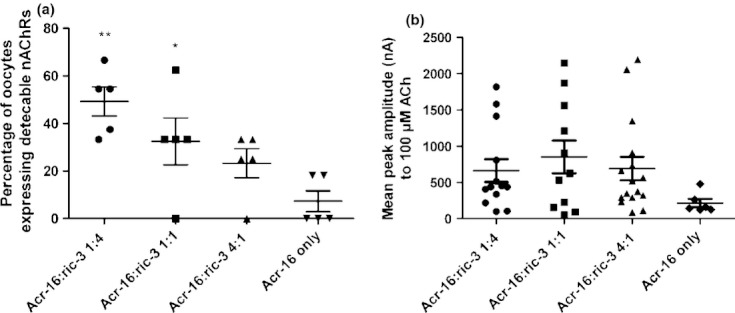
The effect of varying the amount of *X. laevis* RIC-3 on the expression of ACR-16 in *Xenopus* oocytes. (a) The percentage of oocytes expressing detectable currents (> 10nA) in response to 100 μM acetylcholine (ACh) was determined on the experimental weeks in which oocytes from all conditions were screened. The mean percentage expression with the injection of different ratios of *acr-16*:*X. laevis-ric-3* cRNA was as follows; 49 % for 1 : 4, 32 % for 1 : 1, 23 % for 4 : 1 and 7 % for 1 : 0. A One-Way anova with a Tukey's Multiple Comparison Test revealed that the 1 : 4 and 1 : 1 ratio conditions gave significantly more frequent detectable expression than in the 1 : 0 ratio condition (** and * respectively). The 1 : 4 ratio condition was also significantly different to the 4 : 1 condition (*). (b) Mean peak amplitude to 100 μM ACh on ACR-16 expressed in oocytes injected with different ratios of *X. laevis ric-3* cRNA to subunit cRNA showing SEM (*n* = 5, total n for oocytes equal or above 7). Each symbol represents the mean peak amplitude response from a single oocyte on application of 100 μM ACh. The mean results for each condition were as follows; *acr-16*:*X. laevis ric-3* 1 : 4 = 662.5 nA, 1 : 1 = 850.7 nA, 1 : 4 = 690 nA, 1 : 0 = 434.4 nA. A one-way anova found that there was no significant difference between the conditions.

Expression of the ACR-16 nAChR was robust when co-expressed with the *X. laevis* RIC-3, with an EC_50_ for ACh of 24 μM, (95% CI = 19–30 μM), and a Hill Slope of 2.18 (Fig. 2), consistent with previously published data (Raymond *et al*. 2000). The EC_50_ for nicotine was 22 μM (95% CI = 15–33 μM), and a Hill Slope of 1.91. When expressed in the absence of RIC-3, ACR-16 produced very small or undetectable currents in the majority of oocytes, when 100 μM ACh or nicotine were applied; we were not able to measure those currents accurately so they have been discarded in all the subsequent data analyses. However, in a minority of oocytes, ACR-16 expression in the absence of any RIC-3 resulted in substantial ACh-induced currents (Fig. 3).

We tested the effects of varying the relative expression of the *X. laevis* RIC-3, compared with the ACR-16 receptor, by injecting the two cRNAs at a ratio of 1 : 4, 1 : 1 and 4 : 1 *ric-3*:*acr-16*. In each case, the same amount of receptor cRNA was injected into the oocytes. The proportion of oocytes that produced measurable currents in response to application of 100 μM ACh was increased for all *ric-3*:*acr-16* cRNA ratios compared with those cells injected with *acr-16* cRNA alone (Table 2). We produced and compared concentration-response curves for various ratios of *ric-3*:*acr-16* cRNAs injected into oocytes. All the EC_50_ values for acetylcholine were similar (Table 2), regardless of the ratio of *acr-16*:*ric-3* cRNA that was injected into the oocytes and the confidence intervals for the 1 : 1 ratio overlapped with that shown in Fig. 2. In addition, the peak response to application of 100 μM ACh was the same, regardless of the ratio of *ric-3* to *acr-16* cRNAs tested (Fig. 3). The injection of equal amounts of *ric-3* and *acr-16* cRNAs was therefore used in subsequent experiments.

**Table 2:** The amount of *X. laevis* ric-3 cRNA co-injected into the oocytes has no effect on the EC_50_ for acetylcholine at the ACR-16 receptor, but does increase the proportion of oocytes giving detectable currents (> 10nA) in response to application of 100 μM ACh

Ratio *acr-16/X. laevis ric-3*	1 : 0	4 : 1	1 : 1	1 : 4
Proportion of oocytes (%) from which currents > 10nA were detected	7	19	33	49
EC_50_ ACh	35 μM	29 μM	40 μM	42 μM
95% Confidence fInterval	27–45 μM	26–32 μM	19–81 μM	31–56 μM

### A comparison of the effects of RIC-3 from *X. laevis*, *C. elegans* and human on the functional expression of ACR-16 and human α7 nAChR

**Fig. 4:**
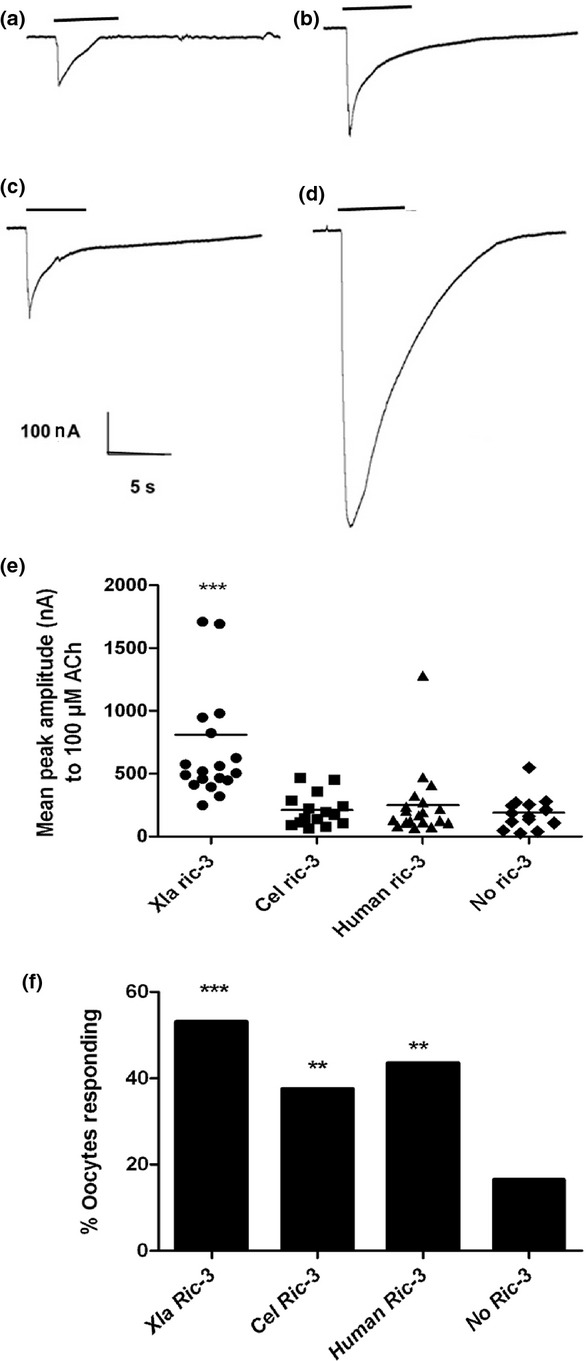
A comparison of the effects of RIC-3 from *Xenopus,* human and *C. elegans* on the expression of ACR-16. Example responses to 100 μM acetylcholine (ACh) from oocytes expressing *C. elegans* ACR-16 (a) in the absence of any RIC-3 and together with RIC-3 from (b) *C. elegans,* (c) human and (d) *X. laevis*. (e). The mean peak current amplitudes to 100 μM ACh recorded from voltage clamped oocytes 48 h post-injection with a 1 : 1 ratio of acr-16 cRNA together with ric-3 cRNA from the three species or with none. Each data point represents a single oocyte, and the combined data were obtained from oocytes isolated from at least four different animals. (f) The proportion of the total oocytes tested from which detectable currents (> 10nA) were evoked by the application of 100 μM ACh. ***p = <0.001, **p = <0.01 as compared with the no ric-3 condition.

We injected oocytes with *acr-16* cRNA together with either *X. laevis,* human or *C. elegans ric-3* cRNA. In each case, equal amounts of the *ric-3* and *acr-16* cRNAs were injected. Co-injection of the *X. laevis ric-3* cRNA with *acr-16* resulted in significantly larger peak currents upon application of 100 μM ACh (Fig 4) than did co-injection of the human or *C. elegans ric-3*. The mean current responses to 100 μM ACh were 854 nA for *X. laevis* RIC-3, 160 nA for *C. elegans* RIC-3, 247 nA for human RIC-3 and 187 nA for ACR-16 alone. The responses from the oocytes injected with *X. laevis ric-3* cRNA were significantly higher than those injected with *C. elegans* or human *ric-3* cRNA, or with none. There were no significant differences in peak amplitude to 100 μM ACh between the *C. elegans*, human and *acr-16* alone conditions, though the proportion of oocytes producing detectable currents was significantly lower in the absence of any RIC-3, which confirms that the human and *C. elegans* proteins were being functionally expressed.

**Fig. 5:**
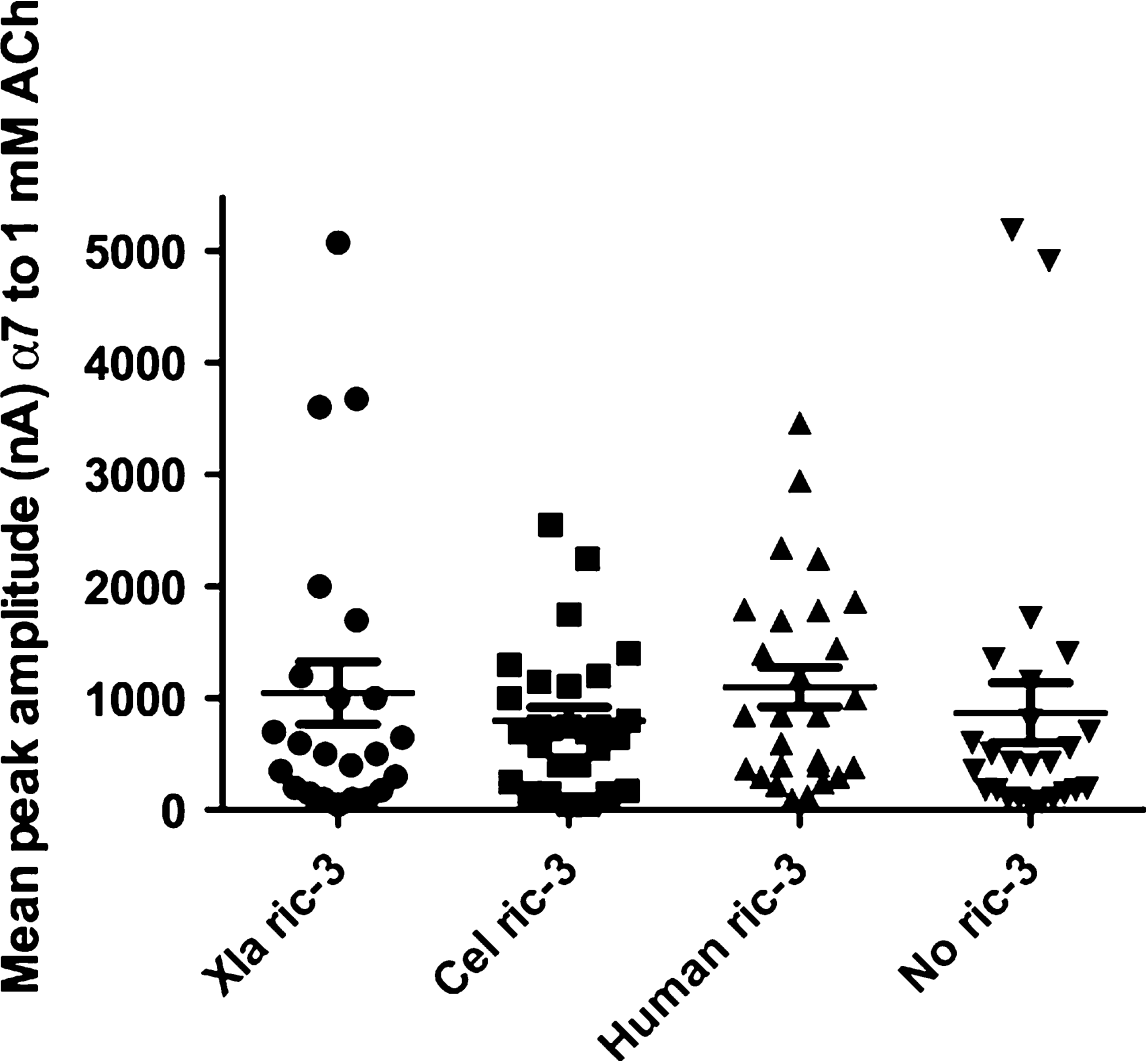
A comparison of the effects of RIC-3 from *Xenopus,* human and *C. elegans* on the expression of human α7. The maximal current evoked by application of 1 mM ACh was measured in the absence of any exogenous RIC-3 and following co-expression with RIC-3 from *C. elegans*, human or *X. laevis* at a 1 : 1 ratio. Each data point represents a single oocyte. No significant differences were detected between the experimental conditions.

As co-expression of the invertebrate ACR-16 receptor with the *X. laevis* RIC-3 led to larger amplitude ACh-induced currents than was achieved with the human or nematode orthologues, we compared the ability of all the three RIC-3 proteins to stimulate expression of the human α7 nAChR. Oocytes were injected with α7 cRNA with or without one of the RIC-3 cRNAs. Interestingly, mean currents detected in response to application of 1 mM ACh were very similar, irrespective of the presence or absence of exogenous RIC-3 (Fig. 5), and a high proportion (> 90%) of oocytes injected with the α7 cRNA expressed currents regardless of whether they were also injected with *ric-3* cRNA or not. No statistically significant differences were observed in the EC_50_ values for ACh from any of these experimental conditions (No RIC-3 = 56 ± 17 μM; + human RIC-3 = 38 ± 7 μM; + *C. elegans* RIC-3 = 65 ± 19 μM; + *X. laevis* RIC-3 = 47 ± 20 μM).

## Discussion

RIC-3 from nematodes, insects and mammals enhances the *in vitro* expression of certain ligand-gated cation channels, including vertebrate α7 nAChR (Halevi *et al*. 2002, 2003; Lansdell *et al*. 2005) and the nematode ACR-16 receptor (Biala *et al*. 2009; Sattelle *et al*. 2009), and RIC-3 is required for the expression of nematode levamisole-sensitive receptors (Boulin *et al*. 2008, 2011). Many of those studies are carried out in the *Xenopus* oocyte system using the mammalian or nematode RIC-3 proteins; however, there is evidence that the activity of RIC-3 is host-cell dependent, with the insect protein being more effective in insect cell cultures and the mammalian protein working better in mammalian cell lines (Lansdell *et al*. 2008). We therefore hypothesized that, for maximum expression in the *Xenopus* oocyte, co-expression with the frog RIC-3 would be optimal. As a first step in testing this hypothesis, we cloned a full length cDNA encoding RIC-3 from *Xenopus laevis*, and found the protein to possess high sequence similarity to human RIC-3 but with two predicted coiled-coil domains, as in the *C. elegans* protein (Halevi *et al*. 2003). *X. laevis* RIC-3 is also predicted to contain two membrane-spanning domains, the first of which is likely to represent a signal peptide.

To test the ability of the *Xenopus* RIC-3 to enhance invertebrate receptor expression in the oocyte system, we used the *C. elegans* ACR-16 nAChR as a model. ACR-16 forms a homomeric nAChR whose expression in the oocyte system is improved by co-expression with RIC-3 (Biala *et al*. 2009). The pharmacology of the ACR-16 nAChR was similar to that described previously (Ballivet *et al*. 1996; Sattelle *et al*. 2009). One apparent difference between the data reported here and that published previously is in the size of the responses produced by ACR-16 expression in the absence of RIC-3; these have been previously reported to be very small (Biala *et al*. 2009). The reason for this apparent discrepancy is that we were not able to reliably quantify the size of the currents in the majority of the oocytes expressing ACR-16 alone, and they have been excluded from the data shown. A proportion (16%) of the oocytes injected with *acr-16* cRNA, but no *ric-3*, produced robust currents and the peak amplitudes of these are presented; this proportion was much lower than in the groups injected with *acr-16* plus any of the *ric-3* cRNAs. These differences were statistically significant (Fig. 4) and demonstrate that the human and *C. elegans* RIC-3 proteins were functional in these experiments. We do not know what allows ACR-16 to be expressed in the absence of the additional RIC-3 chaperone in these few oocytes; it is possible that a small proportion of the oocytes might express low amounts of endogenous chaperones, RIC-3 and others, that permit this expression. Similar ACR-16 currents have been observed by other groups in the absence of RIC-3 (Ballivet *et al*. 1996; Raymond *et al*. 2000).

We found no noticeable difference in amplitude of currents or the dose-response curves in oocytes co-injected with different ratios of *X. laevis ric-3* cRNA alongside the *acr-16* cRNA, and no effects from injecting up to four times more *ric-3* than *acr-16* cRNA. We therefore used a 1 : 1 ratio of *ric-3*:*acr-16* cRNAs, when we compared the ability of RIC-3 from *X. laevis*, humans and *C. elegans* to enhance the expression of ACR-16. Our data indicate that *Xenopus* RIC-3 co-expressed with ACR-16 produced higher current amplitudes in response to 100 μM ACh than human or *C. elegans* RIC-3. We observed no significant difference between the ability of *C. elegans* and human RIC-3 to enhance the expression of ACR-16, suggesting that the species specificity of the chaperone, at least for some receptors, is dependent more on the host cell than on the receptor being expressed (Lansdell *et al*. 2008).

If the *X. laevis* RIC-3 is more effective than the human or *C. elegans* protein in chaperoning the expression of a nematode nAChR, it raises the question of whether this would also apply to mammalian nAChR. As expression of the human homomeric α7 nAChR resulted in robust responses in a high proportion of oocytes, as previously reported (Peng *et al*. 1994), regardless of the addition of exogenous RIC-3, we were unable to determine if these findings could be generalized to vertebrate receptors.

Overall, our data suggest that the *X. laevis* RIC-3 might be a useful chaperone for oocyte expression of invertebrate ligand-gated cation channels and, for certain applications, may have advantages over the use of either invertebrate or mammalian forms of the protein.

## Abbreviations used

ACh: acetylcholine
CI: confidence limits
nAChRs: nicotinic acetylcholine receptors
SOS: standard oocyte saline
